# Biotechnological production of natural pigments for textile dyeing

**DOI:** 10.3389/fmicb.2025.1677799

**Published:** 2025-09-30

**Authors:** Bruna Basto, Nuno R. da Silva, José A. Teixeira, Sara C. Silvério

**Affiliations:** ^1^CEB - Centre of Biological Engineering, Universidade do Minho, Braga, Portugal; ^2^LABBELS - Associate Laboratory in Biotechnology, Bioengineering, and Microelectromechanical Systems, Braga, Portugal

**Keywords:** *Penicillium brevicompactum*, natural pigments, agro-industrial byproducts, alternative media, textile dyeing, sustainability and circular bioeconomy

## Abstract

Global markets, including the pigments sector, have been significantly influenced by the adoption of novel circular bioeconomy policies and increasing concerns about sustainable practices. The natural pigments market is expected to grow considerably due to policies promoting renewable resources and reducing environmental impact. Natural pigments, particularly fungi-derived ones, are increasingly favored for their biodegradability, non-toxicity, and additional biological properties, offering a safer alternative to synthetic dyes. This shift is particularly notable in the textile industry, where concerns over synthetic dyes’ environmental and health impacts have prompted the search for sustainable coloring solutions. This work demonstrates the suitability of agro-industrial byproducts, namely cheese whey and corn steep liquor, as alternative culture medium components for producing natural pigments by *Penicillium brevicompactum*. Furthermore, using corncob as a support for fungal immobilization, under submerged fermentation conditions, resulted in high pigment yields, even when the culture medium was composed of only agro-industrial byproducts. Two different natural pigments’ mixtures were successfully used to dye cotton and linen fabrics, highlighting *P. brevicompactum* pigments’ potential as environmentally acceptable alternatives to synthetic dyes. Overall, this work describes, for the first time, the sustainable production of natural pigments and their potential for textile industry applications.

## 1 Introduction

The adoption of bio and circular economy principles has forced the global markets and industries to move toward sustainability practices. The pigments market is no exception, and these concepts have been crucial, particularly in shaping the natural pigments sector, which is expected to grow up to 2.83 billion euros by 2028 (Market Research, 2022). Policies to promote the use of renewable resources, reduce the environmental impact, and respond to consumer demand for eco-friendly products are driving the growth and development of the natural pigments sector (Sharma et al., 2022).

Pigments obtained from natural sources, such as plants, insects, and microorganisms, are being used as a bio-alternative to synthetic pigments (da Silva et al., 2024). These compounds are characterized by their biodegradability, non-toxicity, and non-carcinogenicity, which makes their use safer, besides the possibility of providing additional biological properties (Cristea and Vilarem, 2006).

The textile industry extensively uses non-renewable, synthetic pigments and dyes. However, rising public and customer concerns about health and environmental impact related to the use of synthetic products have forced the sector to drastic changes, namely, to seek and develop novel natural sources of pigments, and design sustainable products and processes with lower impacts (Methneni et al., 2021; Al-Tohamy et al., 2022; Uddin et al., 2022). Replacing synthetic dyes with natural pigments, particularly fungal-derived ones, has recently been gaining more attention (Silva et al., 2020; Sarkar et al., 2022; Umesh et al., 2023).

Generally, microbial natural pigments are attractive options due to their interesting solubility and stability, and the easy handling and manipulation of microbial cultures (Narsing Rao et al., 2017). Additionally, microbial pigments’ production, whether naturally or by genetically modified organisms, offers several advantages such as quick growth, easy downstream processing, and non-seasonal dependence (Manikprabhu and Lingappa, 2013). Furthermore, compared to the use of other natural sources of pigments, such as plants and insects, microorganisms are exempt from raising issues related to the establishment of endangered species, farmland occupation, climate dependence, and ethical concerns (Downham and Collins, 2000).

Among natural pigments produced by microorganisms, the fungal-derived ones have meaningful industrial potential as alternative natural dyes, mainly due to the fungi’s ability to synthesize pigments with a wide color range that can be developed even in scaled and controlled conditions (Sarkar et al., 2022). Even though their low affinity for textile fibers and the relatively high production costs, which still impair their industrial application, there are already some reports on fungal pigments application in different fabrics dyeing, including pigments produced by *Penicillium* species, as summarized in Table 1 (Rather et al., 2023).

**Table 1:** Examples of pigments-producing *Penicillium* species already used for different textile dyeing.

Microorganism	Pigment	Color	Fabric	Reference
*Penicillium chrysogenum*	Quinone	Deep brown	Wool	Atalla et al. (2011)
*Penicillium italicum*	Quinone	Brown	Wool	Atalla et al. (2011)
*Penicillium minioluteum*	Not identified	Red	Leather	Sudha et al. (2016)
*Penicillium murcianum*	Carotenoid	Yellow	Wool	Hernández et al. (2019)
*Penicillium oxalicum*	Anthraquinone (Arpink red™)	Red	Wool	Sardaryan et al. (2004)
	Quinone	Faint reddish brown	Wool	Atalla et al. (2011)
	Not identified	Greenish yellow, light yellow, and greenish	Cotton	Naz et al. (2021)
*Penicillium purpurogenum* (reclassified as *Talaromyces purpureogenus*)	Azaphilone	Yellow	Cotton yarn	Velmurugan et al. (2010)
	Protein complex	Red	Silk and cotton	Sastrawidana et al. (2016)
	Not identified	Red	Wool	Morales-Oyervides et al. (2017)
	Not identified	Red	Wool and silk	Ahmed et al. (2018)
	Not identified	Not specified	Wool	Verma et al. (2019)
*Penicillium regulosum*	Quinone	Brown	Wool	Atalla et al. (2011)
*Penicillium sclerotiorum*	Not identified	Dark yellow	Cotton	Kallingal et al. (2021)
*Penicillium verruculosum* (reclassified as *Talaromyces verruculosus*)	Polyketide	Red	Cotton	Chadni et al. (2017)
*Penicillium* sp.	Not identified	Grayed-orange and grayed-purple	Woolen yarn and cotton	Suciatmih and Hidayat (2017), Suciatmih and Yuliar (2018a)
	Not identified	Orange-white and yellow	Unspecified cloth	Suciatmih and Yuliar (2018b)
	Not identified	Red	Silk	Naaz et al. (2024)
*Penicillium* spp.	Not identified	Reddish brown	Cotton and silk	Jha et al. (2017)

Despite all the inherent advantages, microbial pigment production is still far from being fully implemented by the industry sector. Challenges remain in achieving efficient large-scale production, namely the high production costs (nearly 30% of the overall production cost) due to expensive feedstocks, and the relatively low yields and poor stability of microbial pigments, which have limited their competitiveness against synthetic dyes (Pitol et al., 2017; Lyu et al., 2022). However, significant progress has been made to overcome these barriers. Advances in fermentation technology have allowed optimization of growth conditions such as nutrient supply [e.g., carbon/nitrogen (C/N) ratio], pH, aeration, and incubation parameters, generally related to cell growth factors (spores, seed, and/or inoculum age) that directly enhance pigment yield and quality (Aman Mohammadi et al., 2022). Furthermore, the adoption of metabolic and genetic engineering strategies has opened new possibilities for tailoring pigment biosynthesis pathways, enabling both increased yields and modification of pigment properties to meet industrial requirements.

Economic feasibility of microbial pigments industrial production can also be improved through the valorization of low-cost substrates. Agro-industrial residues and byproducts, often considered environmental pollutants, are increasingly used as fermentation substrates (Sajjad et al., 2020; Basto et al., 2022). This approach not only reduces the production costs but also contributes to waste management and circular economy principles, further strengthening the environmental sustainability associated with microbial pigments production. Thus, the future of microbial pigments production seems to rely on the integration of advanced cultivation techniques, strain engineering, and sustainable bioprocessing. As these strategies converge, microbial pigments are expected to become cost-effective and reliable on an industrial scale. Their adoption will enable industries to reduce dependence on fossil-derived synthetic dyes while providing safer and multifunctional alternatives (Ramesh et al., 2019, 2022; Chatragadda and Dufossé, 2021). Cheese whey (CW) is one of the main byproducts of the dairy industry, being obtained from the precipitation and removal of milk casein during cheese-making (Zotta et al., 2020). Over 190 million tons of cheese whey are generated globally each year, and approximately 50% of that amount is released into the environment untreated (Asunis et al., 2020; Papademas and Kotsaki, 2020). Currently, CW is mainly used as a feedstock for animal feeding or to produce ricotta cheese. However, this byproduct is rich in lactose, soluble proteins, lipids, and mineral salts, which can be used in fermentative processes to obtain added-value products (Zotta et al., 2020).

In the corn processing industry, corn steep liquor (CSL), a byproduct composed of organic acids, amino acids, vitamins, and sugars, is obtained by steeping corn in water and concentrating the liquid (Nascimento et al., 2009; Maddipati et al., 2011; Kang et al., 2020). Due to its low cost and nutritional composition, CSL is widely used as an alternative nitrogen source in microbial fermentation, being much cheaper than yeast extract (Martinez-Burgos et al., 2021). Corncobs, another agro-industrial waste product with good water retention capacity, are underutilized and could also serve as a low-cost support option for microbial immobilization (Laopaiboon and Laopaiboon, 2012).

Recently, we reported the successful utilization of alternative culture media to produce pigments by *P. brevicompactum* under different fermentation conditions (Basto et al., 2022). In this work, aiming to tackle the issues associated with the high production costs of natural fungal pigments, the agricultural residue corncob was used as a natural organic support for mycelium immobilization, and the pigments’ production by *P. brevicompactum* was studied in alternative low-cost culture media containing CW and CSL. Finally, the coloring capacity of the produced pigments was assessed on different fabrics, for the first time, to determine their technological potential for application in the textile industry.

## 2 Materials and methods

### 2.1 Microorganism

*Penicillium brevicompactum* (MUM 02.07) was sourced from the Mycology collection of the University of Minho (MUM). Spore suspensions [preserved in semi-solid agar medium (2 g L^−1^) at room temperature] were used to prepare fungi’s stock cultures in malt extract agar (MEA) medium [(g L^−1^): malt extract (20), glucose (20), peptone (1), and agar (20)], which were grown for 7 days at 25 °C.

### 2.2 Industrial byproducts

Corn steep liquor (CSL) and cheese whey powder (CW) were kindly provided by COPAM - Companhia Portuguesa de Amidos, S.A. (São João da Talha, Portugal) and Lactogal Produtos Alimentares S.A. (Modivas, Portugal), respectively. The compositions were previously determined and reported as (% w/v) 7.5 sugars and 0.5 proteins for CSL (Gudiña et al., 2015), and (% w/w) 58.5 lactose, 12.6 protein, <0.2 fat, and 1.2 moisture for CW (Basto et al., 2022).

### 2.3 Natural support

Corncobs were collected from a local harvest (Lousada, Porto, Portugal) and cut into pieces with approximately 0.1 cm^3^. The prepared material was soaked in deionized water at 80 °C for 6 h, for washing and to increase porosity, and finally dried at 60 °C (Velmurugan et al., 2011). Before use, the support was autoclaved at 121 °C for 15 min.

### 2.4 Commercial fabrics

Raw, pure cotton and linen fabrics were obtained from a local fabric store located in Arco de Baúlhe, Braga, Portugal. The fabrics were cut into small circles (3.5 cm in diameter) and washed with detergent at 60 °C, except for the non-washed control. After washing, the fabrics’ pieces were dried at 60 °C.

### 2.5 Inoculum preparation

Fungal inocula for fermentations were prepared by adding 1–2 mL of a sterile saline solution (0.85% w/v NaCl containing 0.01% w/v Tween 80) to fully sporulated *P. brevicompactum* stock cultures (Cardoso et al., 2017). Spores were scraped from the agar plates under aseptic conditions, and the conidia suspension (10^6^ conidia mL^−1^) was used as inoculum.

### 2.6 Pigments’ production

Seven distinct culture media were prepared. Table 2 lists those media and their compositions.

**Table 2:** Different media prepared and their corresponding composition.

Medium	Representative icon	Composition (g L^−1^)
A	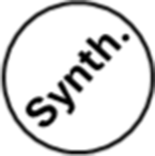	Lactose (20), yeast extract (8), peptone (8), KH_2_PO_4_ (2), Na_2_HPO_4_·12H_2_O (8), MgSO_4_·6H_2_O (0.25)
B	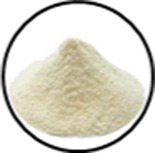	CW (34.6)
C	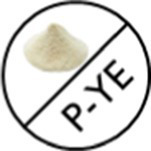	CW (34.6), yeast extract (0.5), and peptone (0.5)
D	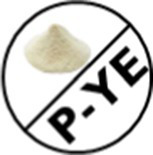	CW (34.6), yeast extract (4), and peptone (4)
E	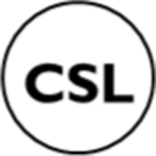	CSL (12.55)
F	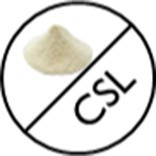	CW (34.6), and CSL (1)
G	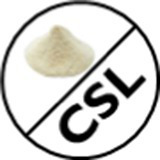	CW (34.6), and CSL (8)
Control	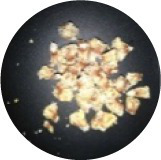	Corncob (7 g) soaked in water

To reduce its color contribution to the culture medium, the maximal CSL concentration in medium C was previously defined as 12.55 g L^−1^ (Basto et al., 2022). All the culture media (A–G) were used for both submerged [with free (SmF) or immobilized mycelium (SmFi)] and solid-state fermentation (SSF) studies. Fermentations were carried out for 12 days in an orbital shaker at 23 °C and 150 rpm. These experimental conditions were previously determined and optimized for pigment production by *P. brevicompactum* grown in a synthetic fermentation medium (Fonseca et al., 2022). Two hundred fifty milliliter cotton-plugged Erlenmeyer flasks were used to perform the fermentation studies, which were performed in triplicate.

#### 2.6.1 Submerged fermentation (SmF and SmFi)

Submerged fermentation was conducted with free (SmF) and with immobilized mycelium (SmFi), in 50 mL of each culture medium (A–G). For SmFi, approximately 20 pieces of natural support (corresponding to 2 g of corncob) were used as immobilization support. In both modes, samples of the fermentation broths were aseptically collected at 0, 3, 5, 7, 9, and 12 days for further analysis.

#### 2.6.2 Solid-state fermentation (SSF)

SSF was performed using corncob pieces as a natural organic support. According to the volume occupied by the pieces in the Erlenmeyer flasks, 75 pieces of corncob, corresponding to 7 g of support, were used. The selected moisture content (%) corresponded to the maximum volume of the culture medium that the corncob pieces could absorb. Equation 1 was used to determine the moisture content (calculated on a wet basis), according to Ahmad and Munaim (2018).


(1) $$\text{Moisture content}=\frac{\text{Mass of liquid component}}{\text{Total mass}\;(\text{liquid}+\text{support})}\times 100\%$$

After a preliminary test (data not shown), the optimal moisture content was found to be 65% w/w, being applied to all the culture media (A–G) used for pigment production under SSF conditions. A control experiment was performed using corncob soaked in water. SSF samples were evaluated only at the beginning (day 0) and the end (day 12) of the fermentation.

### 2.7 Pigments’ recovery

Pigments, produced under the three different types of fermentation, were recovered from the culture media and the immobilized biomass as described by Basto et al. (2022). In summary, SmF and SmFi culture broths were vacuum-filtered and freeze-dried afterward for further analysis. Additionally, for SmFi, the support containing the immobilized biomass was first extracted with 95% v/v ethanol as in Heo et al. (2018) and finally evaporated at 60 °C. Samples from SSF were also treated similarly, but in this case, all the culture flasks’ content was soaked in ethanol.

### 2.8 Pigments’ analysis

#### 2.8.1 Spectrophotometric analysis

Pigments’ production was monitored by measuring the absorbance of the collected fermentations’ samples at 400, 470, and 500 nm, wavelengths of the visible spectrum corresponding to the yellow, orange, and red regions, respectively (Srianta et al., 2016; Kantifedaki et al., 2018).

To compare the pigments’ production under different fermentation conditions, samples collected after 12 days of fermentation were used. These samples were dissolved in 2.5 mL of distilled water, and the absorbance was measured. For SSF samples, the absorbance obtained for the control test was subtracted from the absorbances measured for all the other culture media.

The sum of the absorbances measured at each wavelength (400, 470, and 500 nm) was used to determine the best condition(s) for pigments’ production.

#### 2.8.2 Thin layer chromatography (TLC) analysis

TLC was used to qualitatively analyze the fermentation samples collected. Three microlitre of the sample, with a concentration of 100 g L^−1^, were loaded on a silica plate (Macherey-Nagel, DC-Fertigfolien ALUGRAM SIL G/UV254, Macherey-Nagel GmbH & Co., Düren, Germany) and allowed to separate using a 50:50% v/v water-ethanol mixture as eluent (Fonseca et al., 2022). The plates were exposed to UV light (366 nm) for spot visualization.

### 2.9 Lactose quantification

Lactose concentration was evaluated by HPLC analysis, using a Jasco chromatograph equipped with a refractive index detector (K-2300, Knauer), and a Prevail Carbohydrate ES column (5 μm particle size, 250 mm length × 4.6 mm internal diameter, Alltech, Grace Davison Discovery Sciences, Bannockburn, IL, USA) fitted with a pre-column using the same stationary phase. A 70:30% v/v mixture of acetonitrile and water was used as a mobile phase at 0.9 mL min^−1^. Lactose standards (1–15 g L^−1^) were used to previously prepare a calibration curve. The samples collected during the submerged fermentations (SmF and SmFi) were centrifuged and filtered through a 0.45 μm sterile syringe filter. Twenty Three microlitre were injected and used to monitor lactose consumption during fermentation (Fonseca et al., 2022).

### 2.10 Fabrics dyeing test

Non-washed control and washed fabric samples were used to assess the dyeing capacity of the produced pigments. Two mixtures of pigments were used: (i) pigments produced under SmF conditions with medium A, to serve as the reference, and (ii) pigments produced under one of the best conditions screened (extracellular medium of the SmFi using culture medium D).

The dyeing test was adapted from Velmurugan et al. (2010) and involved: two controls (with and without fabric washing) and two dyeing approaches (with and without mordanting pre-treatment). For the pre-mordanting treatment, the fabrics’ circles were boiled in an aqueous ferrous sulfate solution (10% w/v) for 30 min. Then, the fabrics were squeezed to remove excess mordant solution and rinsed with water before dyeing. For coloring, aqueous solutions (1 g of lyophilized pigments mixture per 50 mL of distilled water) were used to dip fabrics’ pieces at 60 °C for 90 min. Finally, the dyed fabrics’ pieces were rapidly rinsed and dried at 60 °C before color analysis.

### 2.11 Colorimetric analysis

Color analysis was performed with a Minolta colorimeter (Minolta CR 400, Tokyo, Japan), properly calibrated using a standard white tile (EU certified) to determine chromaticity parameters L^*^ (lightness), a^*^, and b^*^.

The cylindrical coordinate C^*^ (chroma), which represents color saturation, was determined using Equation 2, while the total color difference (ΔE^*^) between control and samples was calculated using Equation 3.


(2) $$C^{*}=\sqrt{a^{*2}+b^{*2}}$$

(3) $$\Delta E^{*}=\sqrt{{\left(\Delta L\right)}^{2}+{\left(\Delta a\right)}^{2}+{\left(\Delta b\right)}^{2}}$$

being ΔL = (L^*^ − L_0_^*^), Δa = (a* − a_0_^*^), and Δb = (b^*^ − b_0_^*^), where L_0_^*^, a_0_^*^, and b_0_^*^ are the values for control and L^*^, a^*^, and b^*^ are the color values of the dyed samples.

### 2.12 Statistical analysis

Experiments were performed in triplicate, and the values were expressed as the means and respective standard deviations (mean value ± SD). GraphPad Prism 6.0 (GraphPad Software, San Diego, CA, USA) was used to perform one-way and two-way ANOVA tests to estimate significant differences among samples with a confidence level of 95%.

## 3 Results

### 3.1 Pigments’ production under submerged fermentation without mycelium immobilization (SmF)

**Figure 1:**
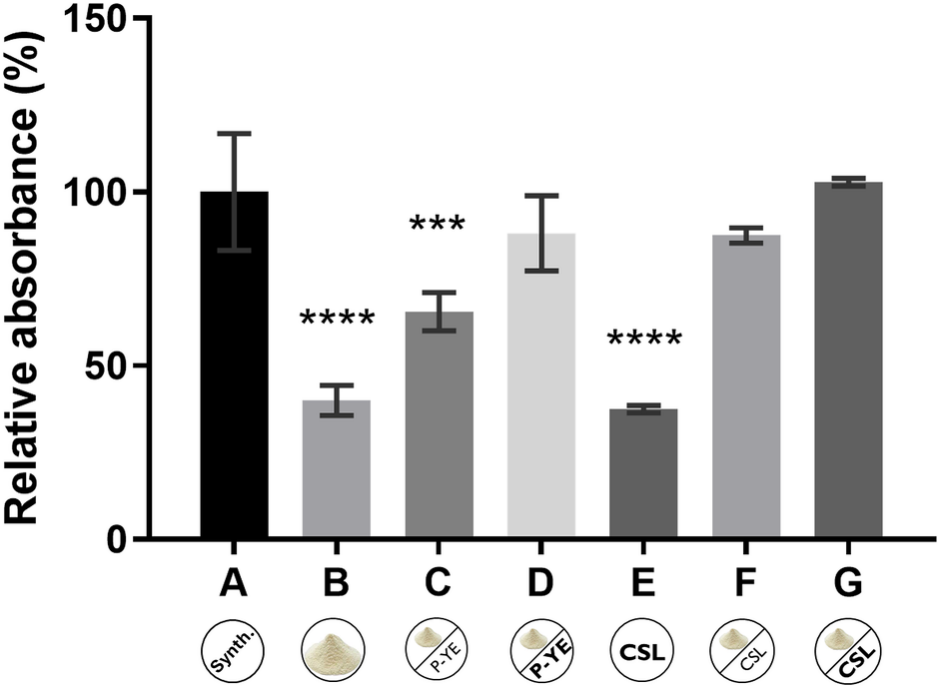
Relative absorbance (%) obtained from the sum of the absorbance values at the three wavelengths (400, 470, and 500 nm) after 12 days of fermentation under submerged fermentation conditions, using the culture media A, B, C, D, E, F, and G. Values are the mean ± SD (*n* = 3). Statistical analysis was performed by one-way ANOVA using Tukey’s multiple comparisons test (^***^p < 0.001 and ^****^p < 0.0001).

**Figure 2:**
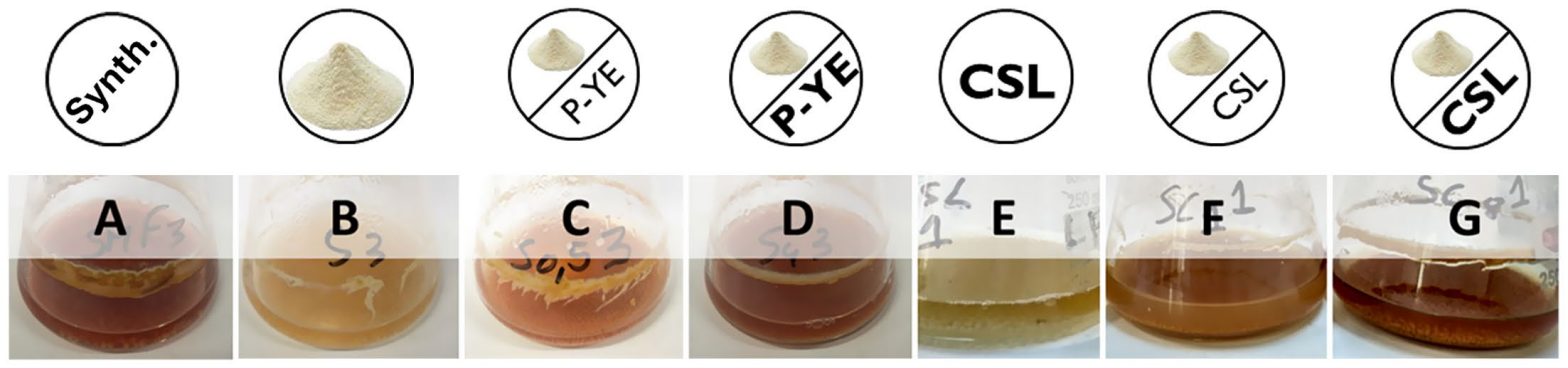
Color differences of the broths after 12 days of submerged fermentation of the respective culture media **(A–G)** under investigation.

The production of the pigments was first investigated under SmF conditions using several alternative culture media (B–G). The results obtained were expressed in relative absorbance (%) and statistically compared with the reference synthetic medium A (Figure 1). Fungal biomass grew in all the culture media tested, indicating that the alternative compositions of the prepared media are suitable substitutes for the synthetic medium A, not causing any impairment to *P. brevicompactum*’s proper growth. Interestingly, the different media compositions induced different colors in both the fermentation broth and the biomass (Figure 2).

There were no statistically significant differences between medium A and media D, F, and G, indicating the suitability of these alternative culture media for pigments’ production by *P. brevicompactum* (Figure 1). It was also observed that medium supplementation with nitrogen and carbon sources resulted in a higher pigment yield (media A, C, D, F, and G). Analyzing the results obtained for media B, C, and D (containing CW supplemented with crescent concentrations of P-YE), it is possible to observe an increase in pigments’ production that follows the increase in the supplements’ concentration. It ranges from 25.5% with medium C to about 48% with medium D, suggesting that under SmF conditions, the concentration of supplements (P-YE) used significantly impacts pigments’ production. When CSL was used as a supplement in the culture media containing CW (media F and G), it led to similar productions of pigments as the reference medium A, even at the lowest CSL concentration (1 g L^−1^). The pigments’ production increase ranged from 47.5% with medium F (lowest CSL concentration), to about 62.8% with medium G (highest CSL concentration) when compared with medium B (no CSL, CW only). Thus, CSL seems to have a positive effect on pigments’ production, leading to a higher increase than P-YE. Additionally, in culture media F and G (with CLS supplementation), color in the fermentation broth was observed as early as the 3rd day, whereas in the others it appeared only on the 5th day.

After 12 days of fermentation, lactose was completely consumed in media A and F. The media C, D, and G presented a lactose concentration lower than 1 g L^−1^ at the final fermentation time. For medium B, the concentration of lactose was higher than 8 g L^−1^. Through the HPLC analysis, it was also possible to verify that medium E had no lactose in its composition.

### 3.2 Pigments’ production under submerged fermentation with mycelium immobilization (SmFi)

**Figure 3:**
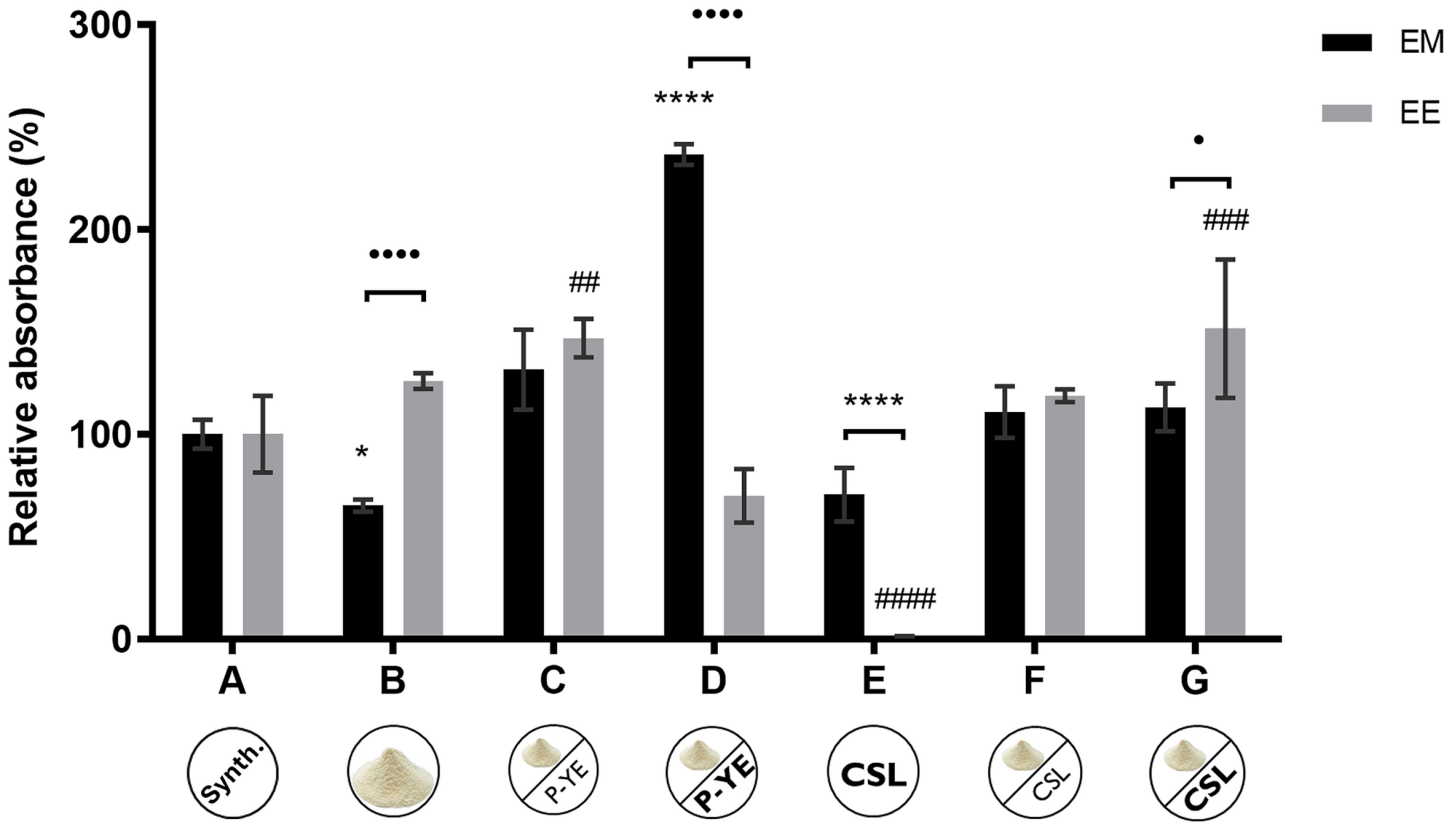
Relative absorbances (%) obtained from the sum of the absorbance values at the three wavelengths (400, 470, and 500 nm) determined for the extracellular medium (EM) and ethanolic extract of the biomass (EE) in each culture media **(A–G)**, under submerged fermentation conditions with mycelium immobilized on corncob (SmFi), after 12 days. Values are the mean ± SD (*n* = 3). Statistical analysis was performed for culture medium samples, ethanolic samples, and between each other by two-way ANOVA (^*^p < 0.05, ^****^p < 0.0001, ^##^*p* < 0.01, ^###^*p* < 0.001, ^####^*p* < 0.0001, ^•^*p* < 0.05, and ^••••^*p* < 0.0001).

Figure 3 shows the results obtained under SmFi for the extracellular media (EM) and the ethanolic extracts of the biomass (EE), expressed in relative absorbance (%). The immobilized biomass showed signs of containing intracellular, or unreleased, pigments due to its coloration (more evident than in the biomass grown under SmF conditions). Thus, it was decided to use an ethanolic solution to extract these pigments from the biomass (EE) and compare their production for all the culture media. Similarly, pigments produced and released to the extracellular medium (EM) were also compared for each alternative medium (Figure 3).

Considering the results obtained for the extracellular medium (EM), similar outcomes were observed when compared with the SmF. Media B, C, and D showed pigments’ production increased with the P-YE concentration used in the culture medium. On the other hand, the supplementation with different concentrations of CSL (media F and G) led to the same increase in pigments’ production. Once again, media B and E presented the lowest production yields, highlighting that pigments’ production drops when the carbon or nitrogen source is absent or limited. Media C, F, and G showed good and equivalent performances to the reference medium A. However, a remarkable result was observed with medium D, whose relative absorbance exceeds 136% of the absorbance obtained with the reference medium (A), indicating that this medium is an effective alternative to the synthetic one.

Regarding the biomass ethanolic extracts (EE), all the media led to equivalent (media B, D, and F) or higher (media C and G) pigments’ productions, except medium E, when compared with the reference medium (A). Significant statistical differences between EM and EE relative absorbances were found for media B, D, E, and G. Higher absorbances, suggesting a higher amount of pigments, were measured in EE fractions of media B and G, while media D and E showed higher values for the EM.

After 12 days, lactose was found to be completely metabolized in media A and D. However, approximately 6 and 4 g L^−1^ remained in the fermentation broth B and F, respectively, while a small amount (approximately 1 g L^−1^) was detected in both media C and G.

### 3.3 Pigments’ production under solid-state fermentation (SSF)

**Figure 4:**
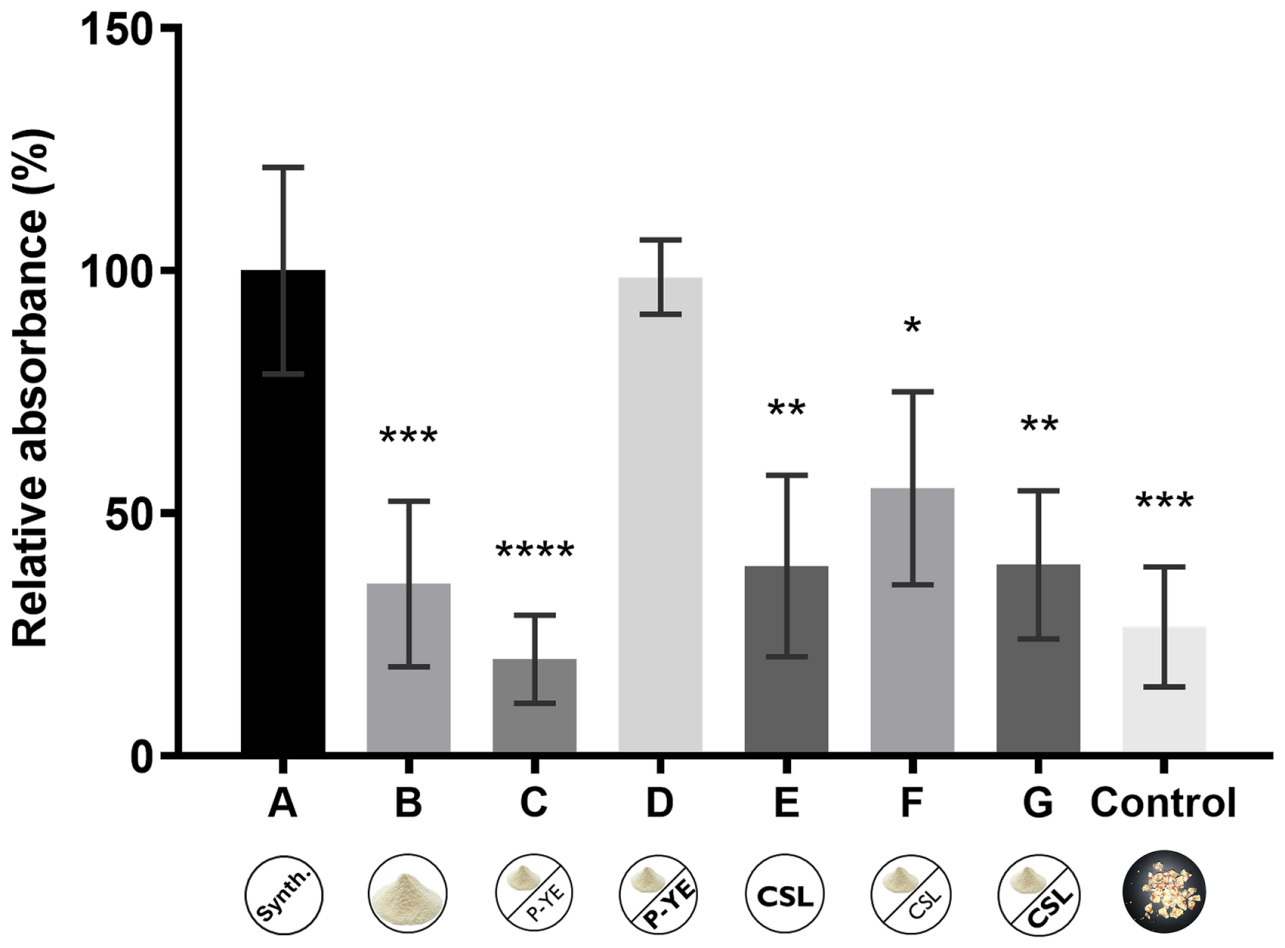
Relative absorbance (%) obtained from the sum of the absorbance values at the three wavelengths (400, 470, and 500 nm) after 12 days of fermentation under solid-state fermentation conditions using corncob as support, and culture media A, B, C, D, E, F, and G. The “Control” bar represents the additional color control where the fungus was grown on corncob soaked only with water. Values are the mean ± SD (*n* = 3). Statistical analysis was performed by one-way ANOVA (^*^p < 0.05, ^**^p < 0.01, ^***^p < 0.001, and ^****^p < 0.0001).

A moisture content of 65% was used in SSF experiments since it was shown to be the maximum amount of culture medium that corncob could absorb. A control experiment with corncob soaked in water (65% moisture content) was also carried out to determine whether corncob could add color to the extract and then interfere with pigment monitoring. It was also examined whether corncob itself could be a suitable substrate for *P. brevicompactum* growth and pigment production. The relative absorbances (%) obtained for the ethanolic extracts of the biomass grown using media A-G are represented in Figure 4. The results showed that only medium D presented a comparable relative absorbance (%) to reference medium A. All the other media presented similar low productions. Also, under SSF conditions, no correlation was found between the increase of nitrogen source supplementation and pigment production when CSL was used (media F and G). However, the culture medium presenting the best results was supplemented with a higher concentration of P-YE (medium D). The use of corncob soaked with water (without any additional nutrients) also promoted some pigment production, despite the low productivity yield obtained (control medium).

### 3.4 Direct comparison of the best conditions for pigments’ production

**Figure 5:**
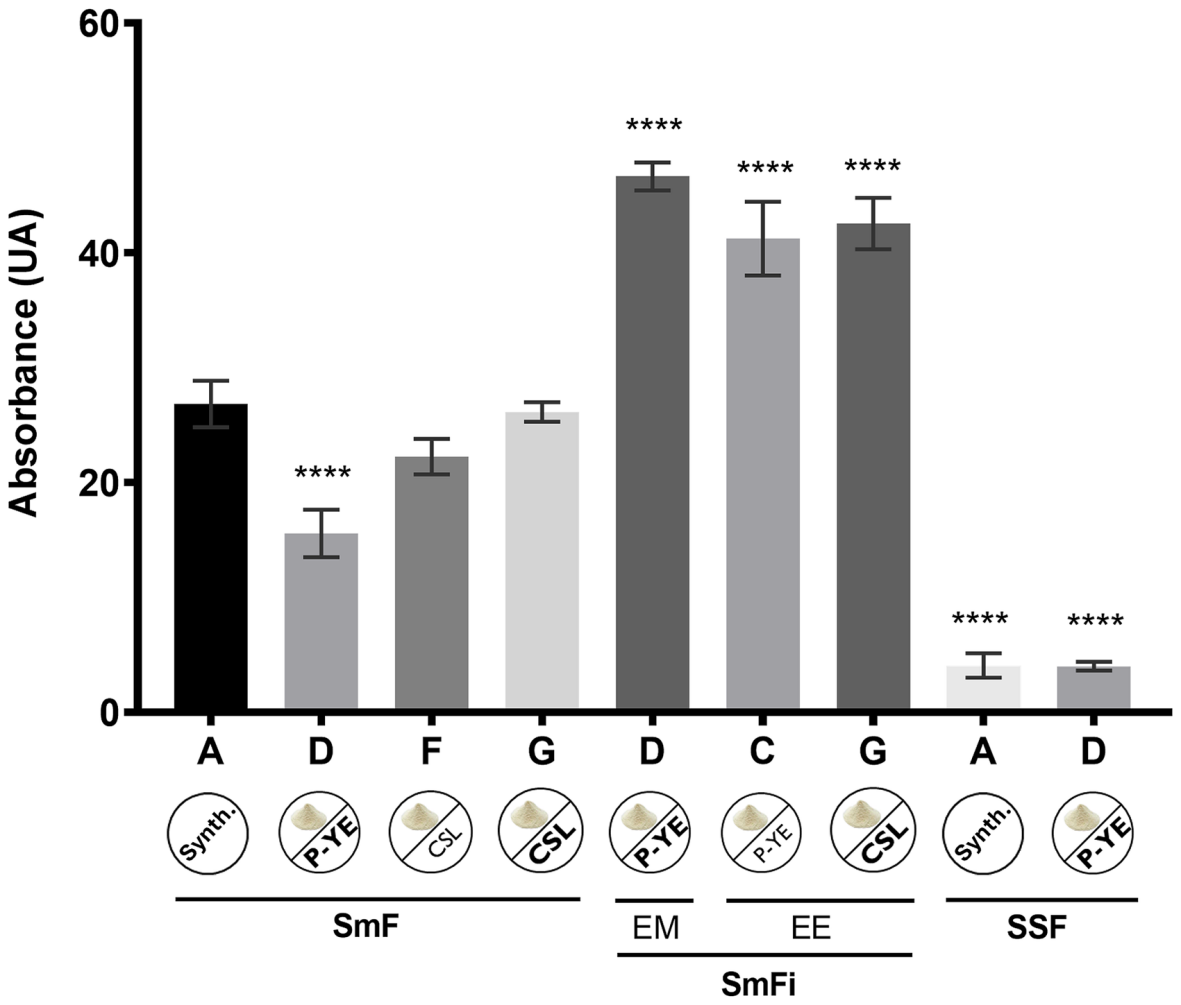
Absolute absorbance values obtained from the sum of the absorbances measured at 400, 470, and 500 nm for the best conditions found under the three studied types of fermentation. (SmF) submerged fermentation; (SmFi) submerged fermentation with mycelium immobilization; (SSF) solid-state fermentation; (EM) extracellular medium; (EE) ethanolic extract of the biomass. Values are the mean ± SD (*n* = 3). Statistical analysis was performed by one-way ANOVA (^****^p < 0.0001).

To better understand the differences in pigments’ production, a direct comparison of the sum of the absolute absorbances was carried out for the best fermentation conditions obtained. Also, this analysis allowed to identify the most suitable conditions to produce pigments with *P. brevicompactum* using low-cost alternative culture media and corncob as a natural immobilization support. The results for the best conditions, for each fermentation type, are summarized in Figure 5.

The data presented in Figure 5 show that among the best conditions selected from each type of fermentation, the very best ones were found under the SmFi conditions (>40 UA), which were 2–10 times higher than SmF and SSF, respectively. Interestingly, according to the medium in use, the highest production of pigments was verified in different fractions (EM or EE). With medium D, a significantly higher amount of pigments was obtained in the EM, while for media C and G, it was found in the EE. To verify whether there were significant differences between the three SmFi conditions, a one-way ANOVA (*p* < 0.05) was performed. The results obtained through this statistical analysis showed no differences between them. Thus, all three media (D, C, and G) provided the best conditions for pigments’ production using *P. brevicompactum* under submerged fermentation with mycelium immobilization on corncob.

### 3.5 Qualitative analysis of the pigment profiles produced

**Figure 6:**
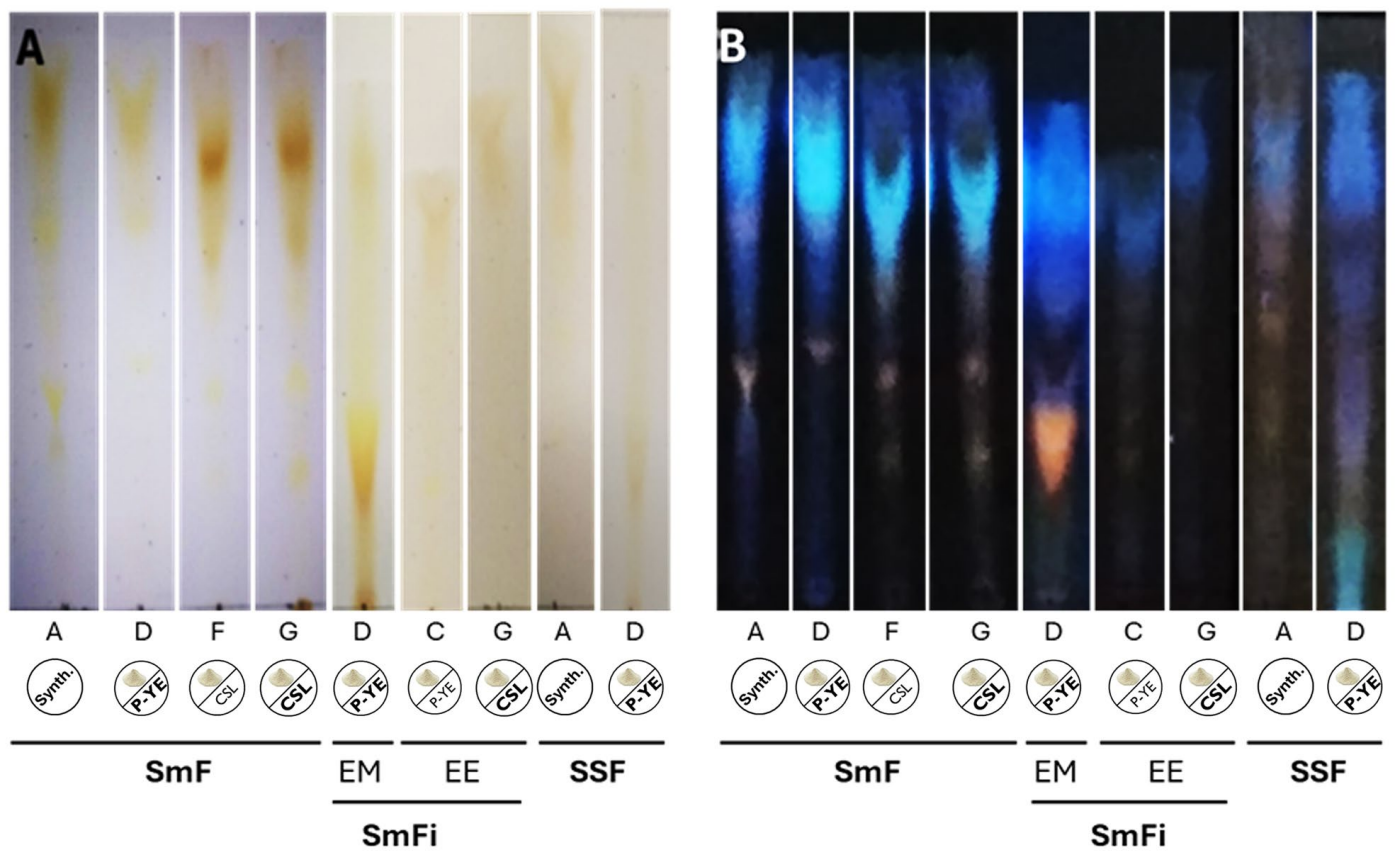
Silica gel TLC showing the separation of the pigments’ mixtures produced under the best conditions (selected for the comparative analysis). **(A)** TLC photographed under visible light; **(B)** TLC exposed at 366 nm.

The pigment profiles were qualitatively examined by TLC to determine if the same mixture of pigments was produced in all the best conditions identified before (Figure 5). The results obtained from this analysis are presented in Figure 6. The chromatographic analysis showed that both the medium composition and the fermentation type can influence the pigments’ mixture formed.

### 3.6 Fabrics’ dyeing and colorimetric analysis

The mixtures of pigments obtained with the reference medium A (free mycelium under SmF conditions) and the alternative culture medium D (EM fraction) from SmFi were used to dye pure and raw cotton and linen to evaluate the possibility of using these pigments in the textile industry.

**Figure 7:**
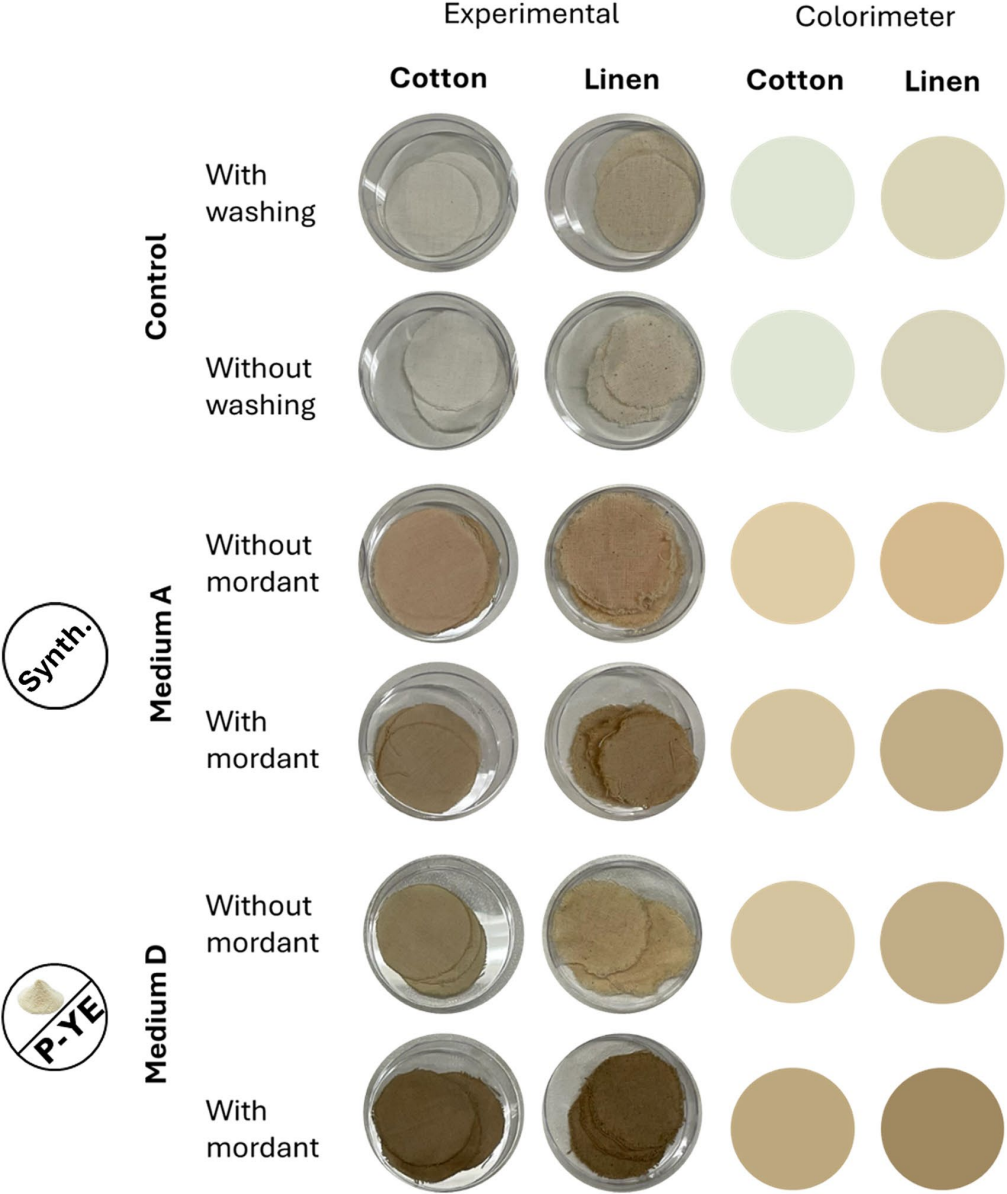
Color visual evaluation (experimental and colorimeter) of the control and dyed fabrics. Colorimeter colors were obtained from Converting Colors CIELab (SAKKOM nteraktiv Ltd., n.d.) using the measured colorimetric parameters.

Visual differences between both dyed fabric samples and the corresponding undyed controls are illustrated in Figure 7.

With the naked eye, it is possible to see the color differences between the two fabrics. Also, the differences are clear between the undyed and the dyed fabrics (with and without mordanting pretreatment). This visual evaluation also showed that treating the fabrics with the mordant agent led to darker, less bright colors. In general, after mordanting pretreatment, the fabrics’ colors got browner. This effect was observed regardless of the pigments’ mixture used for dyeing, even though the medium D pigment mixture developed a darker brownish coloration. Dyeing with medium A pigment mixture showed, in general, a brighter reddish coloration.

The color parameters (L^*^, a^*^, b^*^, C^*^, and ΔE*) of the dyed fabrics were also analyzed, and the results are presented in Tables 3, 4 for cotton and linen, respectively. For both, there are no significant visual differences between the controls since ΔE^*^ is below 2. Conversely, all the dyed samples (with and without mordanting pretreatment) exhibit ΔE^*^ > 16, for both cotton and linen, showing that the pigment mixtures employed to dye the fabrics successfully provided color to them. Worth mentioning that fabrics dyed with the medium D pigment mixture presented higher differences, since their ΔE^*^ values were higher (with and without mordanting pretreatment). The statistical analysis corroborated the conclusions drawn before. For both fabrics, there are no significant differences between the two controls. This means that washing the fabrics did not have any impact on their colors. Thus, it was decided to perform the washing step for the fabrics intended to be dyed. In this way, any impurities present in the fabrics were removed without color loss or changes. On the other hand, when all the dyed fabrics (with and without mordanting pretreatment) were compared with the control (with washing), significant differences (*p* < 0.001 and *p* < 0.0001) were observed for all the color parameters. This validates the visual observation (experimental columns of Figure 7) that both types of fabrics were successfully dyed, with the mixture of pigments from the reference medium A as well as with the mixture of pigments from the alternative medium D. Regarding the dyeing capacity of both pigments’ mixtures, it is possible to conclude, based on the measured color parameters, that fabrics dyed with mixture D are darker (lower L* values) but less deeply and intensely dyed (lower Chroma values). This can be seen in both strategies adopted, with or without fabrics’ mordanting pretreatment.

**Table 3:** Color parameters L^*^, a^*^, b^*^, C^*^, and ΔE^*^ of the cotton fabric obtained for both the controls (with and without washing) and the samples dyed with medium A or D pigment mixtures (with and without mordanting pretreatment).

Color parameters	Control with washing	Control without washing	Dyeing without mordant		Dyeing with mordant	
			Medium A 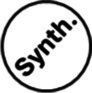	Medium D 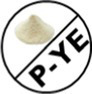	Medium A 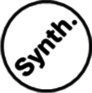	Medium D 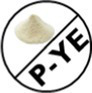
L^*^	90.055	90.355	83.213	79.55^****^	81.18	69.833^***^
a^*^	−4.925	−4.94	0.66	0.227^*^	−0.763	1.213^**^
b^*^	7.805	7.59	21.79	20.807^*^	29.13	24.083^**^
Chroma	9.23	9.06	21.79	20.81^*^	29.15	24.117^**^
ΔE^*^	–	0.369	16.852	17.836	23.782	27.038

(1) Unpaired *t*-test shows no statistical differences between the two controls (with and without washing).

(2) Ordinary one-way ANOVA using Dunnett’s multiple comparisons test shows statistical differences (*p* < 0.0001) between all the dyed cotton samples and the control with washing.

(3) Unpaired *t*-test shows statistical differences between cotton samples dyed with media A and D for each color parameter: ^*^p < 0.05, ^**^p < 0.01, ^****^p < 0.0001.

**Table 4:** Color parameters L^*^, a^*^, b^*^, C^*^, and ΔE^*^ of the linen fabric obtained for both the controls (with and without washing) and the samples dyed with medium A or D pigment mixtures (with and without mordanting pretreatment).

Color parameters	Control with washing	Control without washing	Dyeing without mordant		Dyeing with mordant	
			Medium A 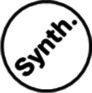	Medium D 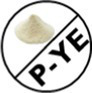	Medium A 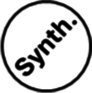	Medium D 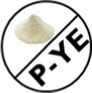
L^*^	84.87	84.685	76.835	71.495^***^	76.05	58.295^****^
a^*^	−2.97	−3	3.395	1.525^**^	1.78	3.055^*^
b^*^	14.88	13.135	25.78	22.005^**^	30.96	24.485^*^
Chroma	15.175	13.47	26.005	22.065^**^	31.01	24.68^*^
ΔE^*^	-	1.755	16.199	16.527	20.375	29.358

(1) Unpaired *t*-test shows no statistical differences between the two controls (with and without washing).

(2) Ordinary one-way ANOVA using Dunnett’s multiple comparisons test shows statistical differences (*p* < 0.0001) between all the dyed linen samples and the control with washing.

(3) Unpaired *t*-test shows statistical differences observed between linen samples dyed with media A and D for each color parameter: ^*^p < 0.05, ^**^p < 0.01, ^****^p < 0.0001.

Our results also showed that dyeing after mordanting pretreatment resulted in a significantly different color (high ΔE^*^ values) for both cotton and linen fabrics compared to the respective undyed controls.

## 4 Discussion

Silvério et al. (2018) observed for the first time the ability of *P. brevicompactum* to produce pigments. Later, Fonseca et al. (2022) successfully showed the production of a mixture of yellow, orange, and red pigments under SmF conditions. In their study, a synthetic culture medium combining lactose with peptone and yeast extract (P-YE) was optimized to achieve the optimal conditions for pigments’ production. Recently, Basto et al. (2022) showed the potential of this fungus to produce diverse mixtures of pigments under different fermentation conditions, using culture media containing agro-industrial byproducts as carbon and nitrogen sources.

The effects of different carbon and nitrogen sources, and their ratios, on fungal growth and pigments’ biosynthesis have been extensively studied and described. Babitha et al. (2007a,b), Gunasekaran and Poorniammal (2008), and Santos-Ebinuma et al. (2013a,b) have shown the impact of using P-YE mixtures as nitrogen sources on different cultures of *Penicillium* strains. Later, Basto et al. (2022) showed that those sources could be successfully replaced by agro-industrial byproducts, such as CW and CSL, still yielding high pigments’ production. Interestingly, in Basto et al. (2022) work, the authors also observed that immobilized fungus in synthetic nylon sponge cubes produced more pigments than the free one, both under submerged and solid-state fermentation conditions.

Fermentation employing natural, local, and abundant residues, wastes, and byproducts allows for energy and resource conservation, as well as the use of alternative low-cost solutions to meet the needs and comply with green policies, circular economy, and sustainable practices (Ramos et al., 2021). These unconventional materials have been explored as alternative substrates for cell growth, but also as supports for biomass immobilization. As substrates, these components are cheaper nutrient sources, and as immobilization supports, they facilitate biomass (and consequently, bioproducts) recovery and reuse. Thus, using these alternative components helps to decrease production costs, making the process cheaper and more attractive (García-Reyes et al., 2017).

Hence, aiming to develop a more cost-efficient, but still suitable, culture medium for pigments’ production, it was decided to use corncob as a natural organic support for mycelium immobilization. Corncob has a good structure and capacity to retain water, making it a potential low-cost support for cell immobilization (Laopaiboon and Laopaiboon, 2012). Furthermore, employing it as a support/substrate offers the option to give this material other uses, decreasing the environmental issues associated with its burning (Hong et al., 2015). In this work, corncob was tested with several culture media supplemented with different ratios of conventional (P-YE) and alternative (CSL) nitrogen sources, containing CW as the main carbon source. Medium A was reported before as an optimized medium for pigments’ production by *P. brevicompactum* under SmF conditions (Fonseca et al., 2022), so it was used in this study as a reference medium. Media B and E, composed only of CW and CSL, respectively, were used as controls for the remaining media supplemented with conventional P-YE mixtures (C and D) or CSL (F and G).

SmF has been widely used at laboratory and industrial scales to produce a variety of added-value bioproducts (García-Reyes et al., 2017). Herein, the pigment production by *P. brevicompactum* under SmF was successfully achieved using alternative culture media based on agro-industrial byproducts (Figure 1), with no negative impact on fungal growth. Moreover, several media (D, F, and G) performed comparably to the synthetic control A. It was also observed that nitrogen and carbon supplementation further enhanced pigment yield. These results matched previously reported observations, where media containing only CW or CSL, led to similar amounts of pigments, corresponding to the poorest performances (Basto et al., 2022). Supplementation with CSL in culture media containing CW (media F and G) resulted in pigment production levels comparable to the synthetic reference medium A, even at the lowest CSL concentration (1 g L^−1^). Compared to medium B (CW only), it was observed that CSL enhances pigment production more effectively than P-YE. Silbir and Goksungur (2019) determined that approximately twice the concentration of CSL is required to obtain the same amount of nitrogen as in a P-YE mixture and CSL. Therefore, the results obtained for media F (1 g L^−1^ CSL) and G (4 g L^−1^ CSL), both with less amount of nitrogen in comparison with media C (0.5 g L^−1^ yeast extract + 0.5 g L^−1^ peptone) and D (4 g L^−1^ yeast extract + 4 g L^−1^ peptone), showed that it is possible to use CSL as an effective alternative supplement. However, it should be emphasized that CSL is a complex residue; thus, some of its additional constituents may also favor fungal growth and consequently pigments’ production. Moreover, it was observed that in media F and G, color appeared in the fermentation broth on the 3rd day of fermentation, while in the remaining media, it appeared only on the 5th day. These findings might indicate that, in these cases, nitrogen depletion and pigment production may be related. By the end of the 12-day fermentation, lactose was completely consumed in media A and F, and nearly depleted in media C, D, and *G. medium* B still had a high lactose concentration, while it was confirmed by HPLC the absence of lactose in medium E. However, it is known that CSL has small amounts of other sugars in its composition, which may contribute to fungal growth (Gudiña et al., 2015). These observations align with the previous work performed by Fonseca et al. (2022), which concluded that lactose is a suitable carbon source for *P. brevicompactum*’s pigment production under SmF conditions.

According to some studies, corncob hydrolysate can be used as a substrate in SmF and as a support/substrate in SSF (Velmurugan et al., 2011; Zhou et al., 2014). Consequently, it was also decided to evaluate all the alternative culture media using the corncob as a natural organic immobilization support (SmFi). Under these conditions, the visibly colored immobilized biomass suggested the presence of intracellular pigments. Thus, these pigments were extracted with ethanol (EE) and compared with the corresponding extracellular production (EM) across all culture media (Figure 3). EM results followed SmF trends, i.e., P-YE (media B, C and D) and CSL (media F and G) enhanced pigment yields, while media B and E showed poor performance due to limited nutrients. Media C, F, and G matched the synthetic medium A, and medium D surpassed it with over 136% absorbance. For EE, excepting medium E, all media performed similarly or better than A highlighting the effectiveness of several alternative media for pigment production under SmFi. Furthermore, different from the results obtained for SmF, EE does not follow the increase in the P-YE supplementation, instead, it seems to be dependent on the rise of CSL concentration.

Ruiz-Sánchez et al. (2023) demonstrated that optimizing the immobilization of *Talaromyces atroroseus* GH2 on an appropriate substrate (nylon sponge) resulted in a 30% increase in pigment production when compared to free cells under submerged fermentation conditions. Additionally, these authors concluded that higher production of pigments was associated with long-term immobilization activity. Our results corroborate the conclusions taken by these authors regarding the advantages of growing the fungus immobilized and demonstrate that corncob presents great potential to be used as immobilization support. The fungus seemed to have adhered to the corncob surface successfully, growing without any impairment.

As corncob showed great potential in SmFi, it was decided to use this natural organic support to produce pigments under SSF. Immobilization provides a feasible approach to improve the stability and reusability of the biomass. Additionally, the downstream processing is facilitated, and the continuous operation mode is easier to implement. Several studies showed that immobilized cultures of fungi can mimic the solid-state environment and effectively improve pigment production compared to liquid-state fermentation (Lyu et al., 2022).

Under SSF, only medium D, supplemented with the highest concentration of P-YE, allowed to obtain pigment production comparable to the reference medium, while other media showed low yields (Figure 4). No correlation was observed between the increasing nitrogen supplementation and pigment production. Interestingly, even the control corncob supported some pigment formation, despite minimal productivity. This finding suggests that this residue could be used not only as a support for mycelium immobilization but also as a substrate for pigment production. However, to increase access to nutrients by the microorganism, further optimization of the growth conditions, and/or specific physicochemical pretreatments of the substrate are needed. In the work of Zhou et al. (2014), it was demonstrated that, following the hydrolysis of the corncob by sulfuric acid and the detoxification of the hydrolysate with calcium oxide, corncob was an effective substitute substrate for *Monascus*’ pigment biosynthesis through submerged fermentation. Also, Morales-Oyervides et al. (2020) obtained a pigment production (16.17 ± 0.37 OD_500_ nm) comparable to the control medium (17.26 ± 0.41 OD_500_ nm) using diluted hydrolysate of corncob treated with acid and without nutrient supplementation.

Our results clearly demonstrated that SSF was not the most suitable fermentation type to produce pigments with *P. brevicompactum* in any of the culture media studied. These results, despite contradicting the fact that SSF resembles the solid-state native environment, which can often improve pigment production, are in line with the observations reported by Rengifo et al. (2023). These authors also compared the ability of different fungi (*Talaromyces brunneus*, *Talaromyces wortmannii*, *Penicillium mallochii*, and *Penicillium maximae*) to produce pigments under SmF and SSF conditions. They concluded that even the same-genus fungi showed different pigment production capacities under different fermentation types. For instance, while *P. mallochii* produced more pigments under SSF conditions, *P. maximae* was unable to produce any pigments. *Talaromyces brunneus* was equally capable of producing pigments under both types of fermentation, yet *T. wortmannii* did not produce pigments under SSF.

To compare pigment production across the best conditions, the sum of absolute absorbances was analyzed, identifying the most suitable low-cost media and fermentation type (Figure 5). The highest pigment yields were obtained under SmFi conditions, reaching production levels around 2 or 10 times higher than those in SmF and SSF, respectively. Medium D (EM) and media C and G (EE) provided the most interesting results. However, it should be emphasized that medium G, entirely composed of byproducts (CW as carbon source, CSL as nitrogen source, and corncob as immobilization support), represents the most attractive condition to produce pigments from a sustainable and economical point of view. CSL was priced at 130 € ton^−1^, representing one-fifth the cost of yeast extract (around 908 € ton^−1^) (Martinez-Burgos et al., 2021). This means that roughly, medium G can be at least 3.5 times cheaper than the media supplemented with the P-YE (C and D). However, this difference is even higher if the peptone (P) price is also considered.

Some studies have been published exploring the potential of *Penicillium* to produce pigments in synthetic media. Santos-Ebinuma et al. (2013a) obtained a sum of absorbances of 6.79 UA for a mixture of yellow, orange, and red pigments using sucrose and yeast extract as carbon and nitrogen sources, respectively, for the growth of *P. purpurogenum* DPUA 1275 under submerged fermentation conditions. De Araújo Alencar et al. (2016) also studied two *Penicillium* strains to produce red pigments, combining different synthetic sources of carbon and nitrogen. The highest sum of the absorbances was 4.36 UA, obtained with glucose and tryptone. In another study, Pandey et al. (2018) used a fungal isolate identified as *Penicillium* sp. to produce an orange pigment by submerged fermentation. They reported a maximum of 3.39 UA using potato dextrose as a carbon source. Recently, Molelekoa et al. (2021) evaluated the production of pigments using a mixture of green wastes (pineapple, papaya, and banana peels) and milk whey as substrates, under submerged and solid-state fermentation conditions. The best results were found under solid-state fermentation with a maximum of 4.00 UA using *Penicillium* strains.

Compared to the published data, our results show that corncob-immobilized *P. brevicompactum* grown in alternative media (such as media C, D, and G) allows a 10 times greater pigment production. Besides, in the case of medium G, the high-yield production of pigments also allows the valorization of byproducts generated from the agro-industrial sector. Thus, not only are more added-value compounds (pigments) being produced, but their production costs are highly reduced, and the developed bioprocess can be considered more environmentally friendly, complying with the circular bioeconomy policies.

On the other hand, the chromatographic analysis indicated that different pigments’ mixtures can be produced depending on the medium composition and the fermentation type. For example, under SmF conditions, media with completely different compositions (A, D, F, and G) showed different pigment profiles. Media F and G showed higher similarity because the only difference in their compositions is the concentration of CSL used (1 and 8 g L^−1^, respectively). Regarding media A and D, changing the carbon source (CW instead of lactose) impacted the pigments’ mixture produced.

Concerning the fermentation type, its impact on the mixture of pigments produced is evident in media A (SmF vs. SSF), D (SmF vs. SmFi vs. SSF), and G (SmF and SmFi). Despite the composition of each medium being maintained, the fungus produced different pigment mixtures in all these cases.

Variations in pigment mixtures produced by the same fungus under different fermentation types have already been reported, as in the work of Rengifo et al. (2023). Such variations are frequently attributed to differences in oxygen availability, water activity, nutrient diffusion, pH and temperature gradients, and stress responses between SSF and SmF conditions. These environmental and operational differences can influence the fungi’s metabolic pathways and secondary metabolite production, leading to distinct pigment profiles (Papagianni, 2004).

Food and beverages are among the most important applications of natural pigments. However, these molecules can be valuable in other industrial sectors. For instance, in the textile industry, natural pigments, particularly fungal pigments, can be promising alternatives to synthetic dyes. Fungal pigments often present great colorfastness and staining properties. They are also known for their high stability and consistency, warranted biodegradability, and production under controlled conditions with no seasonal dependence. The pigment mixture obtained from *P. brevicompactum* using the culture medium A was previously tested for stability across a broad temperature range (5–85 °C) and pH spectrum (3–10) over 24 h, demonstrating full stability under all conditions (Fonseca et al., 2022). Besides, these pigments come from renewable sources, and their use will contribute to reducing the release of toxic substances into the environment, generally associated with synthetic pigments. Additionally, some of these natural pigments can absorb UV light, conferring protection against harmful radiation, which provides an additional benefit to the fabrics dyed with them. Nonetheless, despite the advantages of fungal pigments, they still do not fulfill industry expectations, mainly due to their irregular fixation and the lack of standardized methods for their use in the industrial dyeing process (Venil et al., 2020).

The potential of the application of fungal pigments in the textile industry has already been investigated. Some fungal pigments, produced by species of *Trichoderma* sp., *Drechslera* sp., *Aspergillus* sp., and *Curvularia* sp. were already tested, presenting similar characteristics as conventional synthetic dyes (Roca et al., 2023). In this work, raw and pure cotton and linen were dyed with pigment mixtures obtained from the reference medium A (SmF) and from the alternative medium D (SmFi, EM). Clear color distinctions were observed between dyed fabrics, with or without mordanting pretreatment. Mordant treatment generally resulted in darker, less vibrant tones, shifting the colors toward brown, regardless of the pigment mixture used. Pigments from medium D produced a deeper brownish hue, while those from medium A yielded brighter reddish shades, as supported by the color parameters (L^*^, a^*^, b^*^, C^*^, and ΔE^*^). The Euclidean distance between the CIELAB coordinates of two lights provides a rough guide to their discriminability. The symbol ΔE^*^ is used to denote the distance in the uniform color space (Brainard, 2003). According to Stokes et al. (1992) and Brainard (2003), when the values of ΔE^*^ between two colors are under 2.2, the colors are not discriminably different from each other, and vice-versa. Herein, all dyed samples, whether pretreated with mordant or not, showed ΔE^*^ values above 16, demonstrating effective coloration by both pigments’ mixtures, with medium D producing the highest differences (ΔE^*^ > 20). However, fabrics dyed with medium D pigments appeared darker (lower L^*^) and exhibited lower C^*^, indicating less vivid coloration, regardless of mordanting pretreatment.

Natural dyes are known for their low affinity for textile materials, especially for cellulosic fibers. Using natural dyes on textiles often leads to issues like a limited range of shades and reduced color fastness. The focus has mainly been on using mordants to address these problems. Mordants form complexes with dyes and textile fibers, improving their reactivity, durability, and ability to retain color (Repon et al., 2024). Recently, some studies about fungal pigments dyeing textiles were reviewed, and the implementation of a mordanting treatment was frequently discussed (Venil et al., 2020). Variations in shade and color associated with mordanting treatments were often reported. For instance, in Mary et al. (2023) work, using a mordant improved color impregnation compared to non-treated fabrics. Furthermore, they observed a direct correlation between mordant concentration and the color that pigments conferred on fabrics.

We may assume that regarding both mixtures of pigments produced by *P. brevicompactum*, a pretreatment with a mordant improves their ability to impregnate color in both fabrics tested. The mordanting pretreatment’s positive effect on fabrics’ dyeing with natural fungal pigments has been shown by several authors (Velmurugan et al., 2010; Chadni et al., 2017; Umesh et al., 2023). In their works, ferrous sulfate was also used as a mordant agent, and the pretreatments led to higher affinities of the different fungal pigments under test. Moreover, the authors also reported darker hues after using this strategy. According to them, this color difference may be due to the formation of dye-metal ion complexes on hydroxyl groups of fabrics’ cellulosic fibers. However, further analysis is required to ascertain the possible reasons for these differences.

Furthermore, other key parameters in textile applications, such as lightfastness, wash fastness, and UV thermal stability, should be further evaluated for the fungal pigments herein tested. Generally, fungal pigments can exhibit notable stability, maintaining performance under harsh conditions. Moreover, studies have reported acceptable color fastness to washing, light, and rubbing for fabrics dyed with fungal pigments (Weber et al., 2014; Hinsch et al., 2015; Hinsch and Robinson, 2018; Hernández et al., 2020). It could also be interesting to pair pigment purification with comprehensive fastness testing to additionally support industrial applicability.

Although mordants can help address the problems of natural, fungal pigment dyeing textiles, their environmental impact is not always favorable. Greener alternatives such as bio-mordants and novel fixation methods have been proposed as more sustainable strategies, with potential to improve colorfastness while reducing the ecological impact of the dyeing process. The potential of bio-mordants such as tannins, chitosan, or plant-derived extracts has been evaluated as an attempt to substitute synthetic or chemical mordants, and alternative methods for improving textile coloration with natural dyes have been investigated (Pranta and Rahaman, 2024). Unconventional solutions, such as using plasma technology, supercritical carbon dioxide, ultrasonic, gamma radiation, UV radiation, and microwave energy, are under study to attain greener textile dyeing processes (Repon et al., 2024). Exploring such approaches will be an important step in future work, now that the potential of crude pigment extracts for textile dyeing has been demonstrated.

## 5 Conclusion

This work demonstrated that alternative culture media composed of agro-industrial byproducts can efficiently replace synthetic media in natural pigments’ production by *Penicillium brevicompactum*. It was shown that alternative media constituted by CW and supplemented with a P-EY mixture or with CSL (media C, D, and G, respectively) led to pigment productions comparable to or even higher than those observed with an optimized synthetic medium.

A remarkable performance regarding pigments’ production was observed when *P. brevicompactum* was immobilized in corncob and grown under submerged fermentation conditions (SmFi) with medium G (34.6 g L^−1^ CW and 8 g L^−1^ CSL). This fermentation condition provides the best balance of sustainability and economic effectiveness. The medium is only composed of cheap agro-industrial byproducts, representing a significant reduction in the production cost. Furthermore, the immobilized biomass allows its reuse and facilitates the downstream processing of the pigments.

Lastly, for the first time, the pigments produced by *P. brevicompactum* were shown to have great potential for textile industry applications. Besides offering an environmentally responsible alternative to synthetic dyes, the produced pigments successfully dyed two different fabrics (cotton and linen). Interestingly, even without mordanting pretreatment of the fabrics, the mixtures of pigments presented the ability to dye the textiles. Given the emerging concerns associated with using hazardous mordant agents, these results demonstrate that these pigments can be applied in novel, green-designed, mordant-free, optimized textile coloring processes.

## Data Availability

The original contributions presented in the study are included in the article/supplementary material, further inquiries can be directed to the corresponding authors.
